# Establishment of the Quantitative Analysis of Multiindex in *Euphorbia lathyris* by the Single Marker Method for *Euphorbia lathyris* Based on the Quality by Design Concept

**DOI:** 10.1155/2021/4311934

**Published:** 2021-09-17

**Authors:** Feng Xuehua, Zhou Guangjiao, Tao Ali

**Affiliations:** ^1^College of Pharmacy, Anhui Xinhua University, Hefei 230088, China; ^2^Bozhou Chinese Medicine Institute, Anhui Academy of Chinese Medicine, Bozhou 236800, China

## Abstract

**Methods:**

The influences of methanol proportion, flow rate, column temperature, and injection volume in the mobile phase on the chromatographic resolution of chromatographic peak of euphorbia factor L1 were experimentally studied via Plackett–Burman design, and the key analysis parameters were screened out; the key analysis parameters were optimized through the central composite design, and the chromatographic analysis conditions were established. Euphorbia factor L1 was taken as the internal reference to construct the relative correction factors for L3 and L4 relative to L1, and their contents were calculated, thus realizing the QAMS. Meanwhile, the euphorbia factor L3 and euphorbia factor L4 were determined using the external standard method, and the differences of values measured by the external standard method from the values predicted by the QAMS method were compared, in an effort to verify the accuracy and feasibility of the QAMS method.

**Results:**

The methanol proportion and column temperature in the mobile phase were the key analysis parameters (*P* < 0.05), and the chromatographic conditions were determined as follows. The methanol/water ratio, column temperature, detection wavelength, flow rate, and injection volume were 60 : 40, 30°C, 275 nm, 1.0 mL/min, and 10 *μ*L, respectively. A total of 20 batches of samples were determined by the QAMS method and external standard method; the relative standard deviations (RSDs) of L3 and L4 determination results were less than 2.0%, without any significant difference.

**Conclusion:**

The QbD-based QAMS method can be used to determine the contents of euphorbia factor L3 and euphorbia factor L4 in *Euphorbia lathyris* L., and it is accurate and feasible.

## 1. Introduction

The quality by design (QbD) concept, which first appeared in Q8 released on the International Conference on the Harmonization of Requirements for Registration of Pharmaceuticals for Human Use (ICH), is defined as “a systematic research method with a predefined target, which highlights the understanding of products and processes and process control based on the reliable and scientific quality risk management” [1, 2]. This method aims to strengthen the drug development and reach the expected product performance through the active design. In short, it means the pharmaceutical quality design. In 2005, the American pharmaceutical industry began to talk about QbD. Pfizer, Novartis, and Merck began to try to use QbD for drug development, registration, and market production. In 2006, food and drug administration officially launched QbD [3, 4]. The high-performance liquid chromatography (HPLC) plays a very important role in the pharmaceutical quality control by virtue of its fast, accurate, and reliable characteristics. By applying the QbD concept to the field of HPLC development, the concepts such as key quality attributes and key analysis parameters are introduced, and a reasonable analysis design scheme is acquired via risk assessment, experimental design, and statistical analysis [5, 6].

When it comes to the quantitative analysis of multicomponents by single marker (QAMS) method, one component in the target sample is taken as the internal reference, the relative correction factors between other components and this component are established, and the contents of other components are calculated through the relative correction factors, so as to realize the multicomponents synchronous determination [7–10]. Being able to effectively solve the problems of shortage of reference substances and high detection cost, this method is applicable to the multicomponents quality control of traditional Chinese medicinal materials [11, 12].

As a traditional Chinese medicinal material, *Euphorbia lathyris* L. is the dry mature seed of *Euphorbia lathyris* L. widely distributed or cultivated in Europe, North Africa, Central Asia, East Asia, South America, and North America [13]. Containing multiple chemical components such as diterpenoids, coumarins, steroids, and flavonoids, *Euphorbia lathyris* L. is of extensive pharmacological activities, such as antitumor, antidiarrheic, and anti-inflammatory effects. The diterpenoid compounds separated out of *Euphorbia lathyris* L. exceed 20 species, and its diterpenoids has two frameworks: lathyrane and ingenane [14, 15], where the former mainly contains euphorbia factors L1, L2, L3, L8, and L9, and the latter mainly contains L4, L5, and L6 [16].

The QbD concept was applied to the HPLC method, the key factors influencing the chromatographic resolution of chromatographic peak were screened out through the Plackett–Burman design (PBD), the key analysis parameters were further optimized via the central composite design (CCD), and the QAMS method was established for the euphorbia factors L1, L3, and L4 in *Euphorbia lathyris* L.

## 2. Materials and Methods

### 2.1. Materials

Agilent 1290 Infinity II HPLC (all-duties pump; multisampler; diode array detector), Agilent UltiMate 3000 HPLC (LPG-3400A quaternion pump; PDA-3000 detector), Waters 2695 HPLC (2695 quaternary gradient pump; Waters 2487 dual wavelength UV detector), chromatographic column (Agilent ZORBAX SB-C18, 250 mm × 4.6 mm, 5 *μ*m; GL InertSustain C18, 250 mm × 4.6 mm, 5 *μ*m; Sagix Copsil C18, 250 mm × 4.6 mm, 5 *μ*m), TC-SY-1000 ultrasonic wave extractor (Beijing Tongde Venture Technology Co., Ltd.), CPA225D electronic scale (one hundred thousandth, Germany Sartorius Stedim Biotech), and XPR analytical balance (ten thousandth, Sweden Mettler-Toledo) were used.

Euphorbia factor L1, euphorbia factor L3, and euphorbia factor L4 reference substances (purchased from Baoji Herbest Biotech Co., Ltd., for the quantitative assay), and methanol (TCI AMERICA, chromatographic grade), acetonitrile (TCI AMERICA, chromatographic grade) were purchased, and the water used was ultrapure water.

### 2.2. Solution Preparation

Preparation of reference solution: accurately weighed the reference substances 10.08 mg euphorbia factor L1, 10.05 mg euphorbia factor L3, and 15.05 mg euphorbia factor L4 in the same 100 mL volumetric flask, dissolved them with methanol and diluted to scale mark, and shook well to obtain a mixed reference solution containing 0.1008 mg, 0.1005 mg, and 0.1505 mg of euphorbia factor L1, euphorbia factor L3, and euphorbia factor L4 in every 1 mL of the sample solution.

Preparation of test solution: took appropriate amount of calamansi and pulverized it through No. 2 sieve to obtain medicinal powder. Took about 0.5 g of the powder, accurately weighed it, added 50 mL of 70% methanol aqueous solution, weighed, and ultrasonically extracted (power 250 W, frequency 40 kHz) for 30 min. Then, placed it at room temperature, weighed it, made up the lost weight with 70% methanol aqueous solution, shook well, filtered, and the filtrate was the test product.

### 2.3. HPLC Chromatographic Conditions

Agilent ZORBAX SB-C18 (250 mm × 4.6 mm, 5 *μ*m) was used; detection wavelength was 275 nm, column oven temperature was 20–30°C, flow rate was 0.8–1.2 mL/min, and injection volume was 5–15 *μ*L. Mobile phase was composed of a certain proportion of methanol and water, the methanol ratio was 60–70% in the mobile phase, and other conditions were determined according to the experimental design [17–19].

### 2.4. Plackett–Burman Design

According to the literature and preliminary experiments, taking the ratio of methanol in the mobile phase (A), flow rate (B), column temperature (C), and sample injection volume (D) as the four factors to be investigated, the chromatographic resolution (Y) of the chromatographic peak of euphorbia factor L1 and euphorbia factor L4 were the key attribute quality [20].

### 2.5. PBD Experimental Program and Results

**Figure 1:**
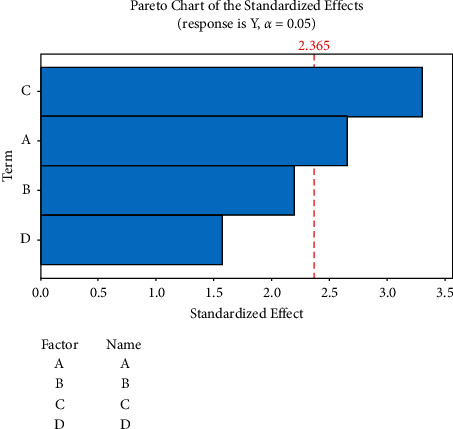
Analysis chart of factor screening Pareto.

Screened the factors with certain risks through PBD and used Minitab to analyze the data [21]. The experimental program and results are given in Table 1, and the factor screening Pareto analysis is shown in Figure 1. The experimental results show that the methanol ratio (A) and column temperature (C) in the mobile phase are significant for the quality of key attributes (*P* < 0.05), which are key analysis parameters, and other factors are noncritical analysis factors, so the flow rate and injection volume are set to 1.0 mL/min and 10 *μ*L, respectively.

## 3. Results and Discussion

### 3.1. Central Combination Design Optimization of Liquid Chromatography Conditions

Based on the PBD experiment, the two key analysis parameters of methanol ratio (X1) and column temperature (X2) in the mobile phase are optimized by CCD, and a mathematical model between it and key quality attributes was established [22], the experimental scheme and results are given in Table 2, and the analysis of variance is given in Table 3.

The *R*^2^ of the Y model is 0.8102, and the regression equation is *Y* = +22.24149 − 0.54921X1 + 0.036763X2 − 0.018600X1X2 + 7.15500E−003*∗*X1^2^ + 0.0218553*∗*X2^2^. From Table 3, it can be seen that the methanol ratio and column temperature have significant effects on the resolution of euphorbia factor L1 (*P* < 0.05), which is consistent with the experimental results obtained by PBD.

### 3.2. Condition Optimization and Prediction

**Figure 2:**
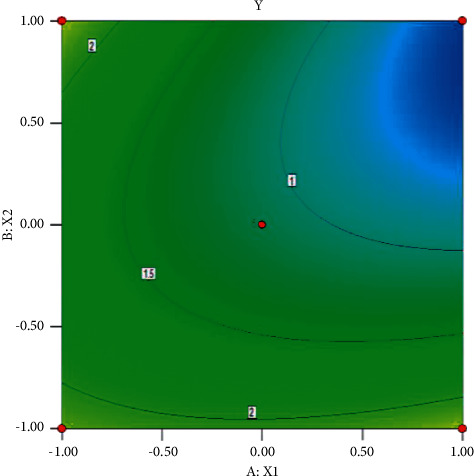
Contour plot of the effect of methanol ratio and column temperature on resolution.

**Figure 3:**
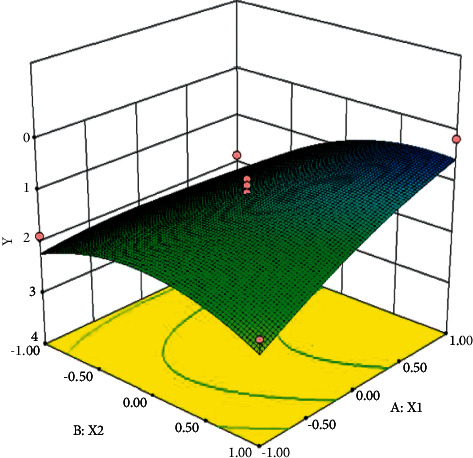
Response surface diagram of the effect of methanol ratio and column temperature on resolution.

The contour plots and response surface plots of each factor to the degree of separation are shown in Figures 2 and 3.

Using Design-Expert.8.0.6 software for derivation, it can be obtained that when the methanol ratio is 60% and the column temperature is 30°C, the resolution can reach 2.33.

### 3.3. Verification Experiment

According to the results of the model analysis, the optimal chromatographic conditions were obtained. The column was Agilent ZORBAX SB-C18, methanol: water (60 : 40), the column temperature was 30°C, the detection wavelength was 275 nm, the flow rate was 1.0 mL/min, and the injection volume was 10 *μ*L. Three samples were tested in parallel, and the average chromatographic resolution was 2.30. The measured value was compared with the value predicted by the equation, and the deviation was calculated. The average deviation was 1.30%, and the deviation was small, indicating that the model was effective.

### 3.4. Study on the Method of “QAMS”

**Figure 4:**
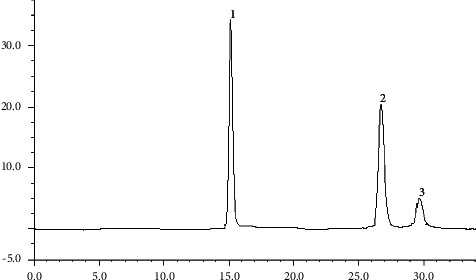
HPLC chromatograms of reference substance. Note: 1, euphorbia factor L3; 2, euphorbia factor L1; 3, euphorbia factor L4.

**Figure 5:**
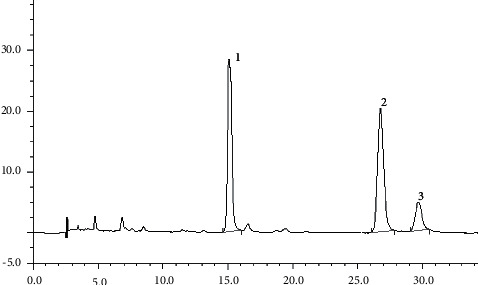
HPLC chromatograms of sample. Note: 1, euphorbia factor L3; 2, euphorbia factor L1; 3, euphorbia factor L4.

According to the chromatographic conditions determined under “3.2,” accurately drew 10 *μ*L each of the mixed reference solution and test solution under “2.2” and injected them into the liquid chromatograph. The results are given in Figures 4 and 5.

#### 3.4.1. Investigation of Linear Relationship

Precisely drew 1, 2, 5, 10, 15, and 20 *μ*L of the mixed reference solution under “2.2,” respectively, and performed regression processing on the peak area integral value based on the injection volume. The results show that each component was within the corresponding range, and the linear relationship was good. The results are given in Table 4.

#### 3.4.2. Stability Test

Took the same test product solution under “2.2,” injected samples after 0, 2, 8, 12, 24, and 36 hours, respectively, recorded the peak area, and calculated the RSD for each component. The RSD values of euphorbia factor L1, euphorbia factor L3, and euphorbia factor L4 were 1.14%, 0.92%, and 1.08%, respectively; all were less than 2.0%. The results showed that the test solution was basically stable after being placed for 36 h under experimental conditions.

#### 3.4.3. Repeatability Test

Took the same batch of euphorbia factor medicinal materials to prepare 6 test product solutions according to the test product preparation method under “2.2,” determined according to law, and the RSD contents of euphorbia factor L1, euphorbia factor L3, and euphorbia factor L4 were 0.99%, 1.02%, and 1.13%; all were less than 2.0%, indicating that the method has good repeatability.

#### 3.4.4. Precision Test

Took the mixed reference solution under “2.2” to repeatedly inject for 6 times, measured the peak area, and calculated the RSD. The RSD values of euphorbia factor L1, euphorbia factor L3, and euphorbia factor L4 are 1.24%, 1.08%, and 1.15% , respectively; all were less than 2.0%, indicating that the precision of the instrument is good.

#### 3.4.5. Sample Recovery Test

Took an appropriate amount of 6 parts of calamansi powder with known content, each about 0.5 g, accurately weighed them, added the same amount of mixed reference solution, respectively, and prepared the test solution according to the method under “2.2,” determined according to law, and the recovery rate was calculated. The average recovery rates of euphorbia factor L1, euphorbia factor L3, and euphorbia factor L4 were 100.14%, 99.97%, and 99.68%, respectively, and the RSD values were 1.02%, 1.06%, and 0.98%, respectively; all were less than 2.0%, indicating that the accuracy of this method is good.

(1) $$\begin{array}{c} \text{Recovery}\left(\%\right)=\frac{\left(A-B\right)}{C}\times 100\%, \end{array}$$

where *A* is the measured value, *B* is the measured component contained in the test sample, and *C* is the amount of control sample added.

### 3.5. Determination of Relative Correction Factor

#### 3.5.1. Calculation of Relative Correction Factor

According to the chromatographic conditions determined under “3.2,” accurately drew the mixed reference solution prepared under “2.2” and injected 1, 2, 5, 10, 15, and 20 *μ*L to determine the peak area of each component. Took the euphorbia factor L1 as the internal standard to calculate the relative correction factors between euphorbia factor L3, euphorbia factor L4, and euphorbia factor L1 according to the relative correction factor calculation formula, namely, fsi = fs/fi = (As/Cs)/(Ai/Ci) (where As is the peak area of the internal reference, Ai is the peak area of other components, Cs is the internal reference concentration, and Ci is the concentration of other components) [23–25], as given in Table 5.

#### 3.5.2. Reproducibility Investigation of Relative Correction Factor

Effect of different brands of chromatographic columns on *f*: according to the HPLC chromatographic conditions determined under “3.2,” accurately drew the mixed reference solution 10 *μ*L prepared under “2.2” and investigated the effects of three different brands of chromatographic columns on the relative correction factors of each component by Agilent HPLC [26]. The results are given in Table 6, which shows that the relative correction factors obtained by different chromatographic columns are basically the same.

Effect of different brands of HPLC instrument on *f*: according to the HPLC chromatographic conditions determined under “3.2,” accurately drew the mixed reference solution 10 *μ*L prepared under “2.2” and investigated the effects of 3 different brands HPLC instrument of UltiMate 3000, Waters 2695, and Agilent 1290 Infinity II on the relative correction factor. The results are given in Table 7. It shows that the relative correction factors obtained by using different brands of HPLC instrument are basically the same.

#### 3.5.3. Positioning of Chromatographic Peaks

It is a problem that must be solved in this method to quickly and accurately identify the other two components in medicinal materials when only one reference substance is used [27,28]. Introduce the relative retention value of each component to be tested as the positioning parameter and investigate the repeatability under the conditions of different brands of instruments and different specifications of chromatographic columns [29]. According to the HPLC chromatographic conditions determined under “3.2,” accurately drew the mixed reference solution 10 *μ*L prepared under “2.2” and determined the relative retention time of the internal reference euphorbia factor L1. According to the relative retention time, the accurate peak positions of the target peak euphorbia factor L3 and euphorbia factor L4 can be correctly judged, the relative retention time error is controlled within 5%, and the results are given in Table 8. It can be seen from Table 8 that the RSD values were all less than 2%, indicating that the relative retention time is used to locate the chromatographic peak and the method is feasible.

### 3.6. Comparison of the Results of the QAMS and the External Standard

Took different batches of medicinal materials, used the external standard method and the QAMS method to calculate the amount of euphorbia factor L1, euphorbia factor L3, and euphorbia factor L4 in *Euphorbia lathyris* L., and the results obtained by the two methods were compared to verify the accuracy of the QAMS method for the content evaluation of multiindex ingredients in *Euphorbia lathyris* L. The results are given in Table 9, which suggested that the results measured by the QAMS method and the external standard method of the euphorbia factor L1, euphorbia factor L3, and euphorbia factor L4 were basically the same, and the SD value of QAMS is small, indicating that the established QAMS method was credible.

## 4. Discussion

The content of euphorbia factor L1, euphorbia factor L3, and euphorbia factor L4 is relatively high in *Euphorbia lathyris* L., and its anticancer biological activity is strong, so it is of great significance to select diterpenoids as the internal quality evaluation indexes for the quality control of *Euphorbia lathyris* L. In this experiment, based on the QbD to design and screening key analysis parameters, a QAMS method for diterpenoids was established. At the same time, the durability of the instrument and chromatographic column was investigated [30–32]. The results show that the experimentally established relative correction factor *f* has good reproducibility, and there is no significant difference between the results obtained by the QAMS method and the external standard method. The QAMS method established in this experiment has high reproducibility, stability, and reliability when determining the amount of euphorbia factor L1, euphorbia factor L3, and euphorbia factor L4, which enriches the quality evaluation method of the content of *Euphorbia lathyris* L., and has provided a more sufficient reference and basis for the promotion and application of QAMS technology in the quality control of traditional Chinese medicine. QAMS is expected to solve the problems of shortage of reference materials and high testing costs in the multiindex quality evaluation of traditional Chinese medicine [33,34].

## Figures and Tables

**Table 1:** PBD experimental program and results.

No.	Methanol ratio A (%)	Flow rate B (mL/min)	Column temperature C (°C)	Sample injection volume D (*μ*L)	Chromatographic resolution Y
1	60 (−1)	1.2 (+1)	20 (−1)	5 (−1)	1.62
2	70 (+1)	1.2 (+1)	30 (+1)	5 (−1)	0.89
3	60 (−1)	0.8 (−1)	30 (+1)	15 (+1)	1.53
4	60 (−1)	1.2 (+1)	30 (+1)	15 (+1)	0.85
5	60 (−1)	0.8 (−1)	20 (−1)	15 (+1)	3.23
6	70 (+1)	0.8 (−1)	30 (+1)	15 (+1)	0.78
7	70 (+1)	1.2 (+1)	20 (−1)	15 (+1)	1.45
8	70 (+1)	0.8 (−1)	20 (−1)	5 (−1)	2.88
9	70 (+1)	0.8 (−1)	30 (+1)	5 (−1)	0.91
10	60 (−1)	0.8 (−1)	20 (−1)	5 (−1)	3.12
11	70 (+1)	1.2 (+1)	20 (−1)	15 (+1)	0.86
12	60 (−1)	1.2 (+1)	30 (+1)	5 (−1)	1.94

**Table 2:** CCD experimental program and response value.

No.	Methanol ratio X1 (%)	Column temperature X2 (°C)	Chromatographic resolution Y
1	65 (0)	25 (0)	1.56
2	65 (0)	25 (0)	1.12
3	65 (0)	25 (0)	0.98
4	72.07 (1.414)	25 (0)	1.33
5	65 (0)	17.93 (−1.414)	3.19
6	65 (0)	25 (0)	0.86
7	65 (0)	25 (0)	1.11
8	60 (−1)	20 (−1)	1.87
9	65 (0)	32.07 (1.414)	1.96
10	60 (−1)	30 (+1)	2.08
11	70 (+1)	20 (−1)	1.84
12	57.93 (−1.414)	25 (0)	2.35
13	70 (+1)	30 (+1)	0.19

**Table 3:** CCD Analysis of variance.

Type	SS (sum of squares)	MS (mean square)	*F* value	*P* value (prob > *F*)	Df	Significance
Model	5.7	1.14	5.97	0.0182	5	Remarkable
X1	1.41	1.41	7.41	0.0292	1	Remarkable
X2	1.26	1.26	6.62	0.0368	1	Remarkable
X1X2	0.86	0.86	4.53	0.0708	1	
X1^2^	0.22	0.22	1.17	0.3159	1	
X2^2^	2.08	2.08	10.88	0.0131	1	
Residual	1.34	0.19			7	
Degree of dissimilarity	1.06	0.35	5.01	0.0768	3	Not significant
Pure error	0.28	0.07			4	
Total difference	7.04				12	

**Table 4:** Standard curve of euphorbia factor L1, L3, and L4.

Compound	Standard curve	*r*	Linear range (*μ*g)

Euphorbia factor L1	*Y* = 1244.6*X* + 10.218	0.9999	0.1008–2.016

Euphorbia factor L3	*Y* = 1516.8*X* − 12.176	0.9998	0.1005–2.010

Euphorbia factor L4	*Y* = 597.4*X* + 11.613	0.9999	0.1505–3.010

**Table 5:** Relative correction factor of two components.

Injection volume (*μ*L)	*f*_L3/L1_	*f*_L4/L1_
1	0.992	1.802
2	0.978	1.791
5	0.994	1.806
10	0.971	1.835
15	0.986	1.794
20	0.989	1.753
Average	0.985	1.797
RSD%	0.90	1.48

**Table 6:** Effect of different brands of chromatographic columns on f.

Chromatographic columns	*f*_L3/L1_	*f*_L4/L1_
ZORBAX SB-C18	0.991	1.802
GL InertSustain C18	0.978	1.813
Sagix Copsil C18	0.984	1.798
Average	0.984	1.804
RSD%	0.66	0.43

**Table 7:** Effect of different brands of HPLC instrument on *f*.

Instrument	*f*_L3/L1_	*f*_L4/L1_
UltiMate 3000	0.980	1.807
Waters 2695	0.979	1.818
Agilent 1290 Infinity II	0.992	1.810
Average	0.984	1.812
RSD%	0.74	0.31

**Table 8:** Relative retention time measured by different brands of instruments and chromatographic columns.

Instrument	Chromatographic columns	Relative retention time	
		Euphorbia factor L3/euphorbia factor L1	Euphorbia factor L4/euphorbia factor L1
UltiMate 3000	Sagix Copsil C18	0.5591	1.1251
UltiMate 3000	ZORBAX SB-C18	0.5671	1.1302
Waters 2695	Sagix Copsil C18	0.5904	1.1260
Waters 2695	GL InertSustain C18	0.5681	1.1311
Agilent 1290 Infinity II	ZORBAX SB-C18	0.5802	1.1265
Agilent 1290 Infinity II	GL InertSustain C18	0.5719	1.1235
Average		0.5728	1.1271
RSD%		1.92	0.26

**Table 9:** Determination of euphorbia factor L1, euphorbia factor L3, and euphorbia factor L4 in *Euphorbia lathyris* L.

Batches	Euphorbia factor L1 (%) (mean ± SD)	Euphorbia factor L3 (%)		Euphorbia factor L4 (%)	
		QAMS (mean ± SD)	External standard method (mean ± SD)	QAMS (mean ± SD)	External standard method (mean ± SD)
20200405 (*n* = 3)	0.2021 ± 0.0079	0.2901 ± 0.0056	0.2899 ± 0.0109	0.1675 ± 0.0083	0.1676 ± 0.0107
20200419 (*n* = 3)	0.3232 ± 0.0102	0.2684 ± 0.0067	0.2687 ± 0.0070	0.1743 ± 0.0068	0.1743 ± 0.0078
20200602 (*n* = 3)	0.2682 ± 0.0072	0.2767 ± 0.0085	0.2768 ± 0.0089	0.1834 ± 0.0087	0.1835 ± 0.0093
20200617 (*n* = 3)	0.1355 ± 0.0087	0.1897 ± 0.0086	0.1899 ± 0.0088	0.1737 ± 0.0102	0.1738 ± 0.0098
20200912 (*n* = 3)	0.2682 ± 0.0110	0.3322 ± 0.0104	0.3320 ± 0.0097	0.2011 ± 0.0096	0.2009 ± 0.0106
20200926 (*n* = 3)	0.3127 ± 0.0067	0.2798 ± 0.0113	0.2797 ± 0.0109	0.1932 ± 0.0078	0.1930 ± 0.0093
20201218 (*n* = 3)	0.2356 ± 0.0093	0.2669 ± 0.0098	0.2667 ± 0.0111	0.1736 ± 0.0069	0.1735 ± 0.0076
10101222 (*n* = 3)	0.3823 ± 0.0095	0.2638 ± 0.0087	0.2637 ± 0.0094	0.1865 ± 0.0109	0.1866 ± 0.0099

## Data Availability

The data used to support the findings of this study are available from the corresponding author upon request.
